# HIV/AIDS, parasites and co-infections: publication patterns in China

**DOI:** 10.1186/1756-3305-2-31

**Published:** 2009-07-09

**Authors:** Li-Guang Tian, Peter Steinmann, Jia-Xu Chen, Shao-Hong Chen, Xiao-Nong Zhou

**Affiliations:** 1 National Institute of Parasitic Disease, Chinese Center for Disease Control and Prevention (China CDC), 207 Rui Jin Er Road, Shanghai 200025, PR China; 2 Department of Public Health and Epidemiology, Swiss Tropical Institute, PO Box 4002 Basel, Switzerland

## Abstract

**Background:**

Since its discovery, HIV/AIDS has arguably captured more attention among the Chinese biomedical research community than most other infectious diseases. Traditional parasitic diseases, on the other hand, are perceived as being increasingly neglected. However, it has long been recognized that interactions between HIV and other infective agents, including parasites, influence the health status of people living with HIV/AIDS. This study aimed at systematically reviewing the Chinese scientific literature on HIV/AIDS and parasites between 1986 and 2006 in order to substantiate or refute these claims, and to highlight neglected research areas.

**Results:**

Searching the three largest Chinese scientific literature databases, in the China National Knowledge Infrastructure (CNKI) a total of 24,511 citations dealing with HIV/AIDS and 15,398 parasite-specific publications were identified. Wanfang Data and VIP Information (VIP) contained 15,925 and 13,873 entries dealing with HIV/AIDS respectively, while 12,043 and 7,068 hits were scored when searching for parasitological references. The number of publications dealing with HIV/AIDS in China increased exponentially from 6 in 1986 to 3,372 in 2006 whereas the publication activity in the field of parasitology was more erratic and lately started to decline. Epidemiology was the most-reported field of endeavor, accounting for 26.0% and 24.6% of the HIV/AIDS and parasitological literature, respectively, while publications dealing with health education only represented 2.9% and 0.7% of all publications, respectively. The total number of Chinese articles focusing on HIV/AIDS and parasite co-infection was 650, with large year-on-year differences in publication numbers. The single-most frequently studied system was HIV-*Pneumocystis carinii *co-infection.

**Conclusion:**

The present study revealed that in China, the fields of parasitic diseases, especially opportunistic parasitic infections linked with HIV/AIDS, is increasingly neglected. This suggests a need to enhance research in the field of opportunistic parasitic infections and parasitology in general.

## Background

It usually takes years after an infection with the Human Immunodeficiency Virus (HIV) for Acquired Immunodeficiency Syndrome (AIDS) to develop. The progressing decline and ultimate collapse of immune system functions which are characteristic for AIDS usually result in morbidity and ultimately death due to opportunistic bacterial, viral and parasitic infections and other conditions. Since the first AIDS case was diagnosed in Los Angeles in 1981 [1], HIV has been detected in virtually every country. The pandemic has resulted in the current infection of an estimated 40.3 million people and has cost at least 25 million lives so far, orphaned millions of children and left scores of people dependent on life-long medication [2,3].

In 1985, the first AIDS case in China was diagnosed at the Beijing Union Medical College Hospital [4]. Originating from several foci, the infection spread throughout the country over the following years and in 2008, approximately 700,000 people were estimated to be HIV positive in China [3]. However, there is a high degree of uncertainty in this estimate since up to 30 September 2008, only 264,302 HIV infections and 34,864 deaths due to AIDS had been officially registered. Chinas position regarding HIV/AIDS markedly changed as the epidemic unfolded. After a phase of neglect, HIV/AIDS received considerable attention from various government sectors due to its apparent potential to develop into a serious public health problem. Different government agencies started to invest significant amounts of money and manpower into HIV/AIDS research, prevention, treatment and care. Currently, free voluntary HIV counseling and testing (VCT) is offered to people at high risk of infection, and free antiretroviral therapy is provided to people living with AIDS according to the national strategy 'Four Free and One Care'. The scheme also includes free education for orphaned children of AIDS victims, and free medical care for people living with HIV/AIDS [5].

The Chinese research community soon became involved in HIV/AIDS research and quickly expanded its activities in the field as official recognition and encouragement were offered, and more dedicated funds were made available. In line with these developments and wider trends in research, the number of scientific publications about HIV/AIDS in China multiplied both domestically and in the international scientific literature. Today, the available domestic Chinese literature is diverse and broad in scope and approaches, and the growth in the number of publications is unabated.

Parasitic infections are still common in many regions and populations across China, and represent a lasting public health challenge. The results of the "National sampling survey of the current situation of major human parasitic diseases in China", which had been implemented between 2001 and 2004 throughout the country, suggest that the overall prevalence of common soil-transmitted helminth infections across China was 21.7%, and that 7.9% of the targeted population segments tested sero-positive for toxoplasmosis. Other parasites were also commonly encountered [6] and additional parasites are emerging or are newly described in China [7,8].

It has long been recognized that interactions between the HI virus and the host facilitate the colonization of the latter by other infective agents, including parasites. This negatively impacts on the health status and outlook of people living with HIV/AIDS. With the progressing development of AIDS, especially once CD4+ T-lymphocyte counts have fallen below 200/mm^3 ^[4], patients often become co-infected by viruses, bacteria or parasites. Such co-infections generally are the proximate cause of death of AIDS patients [9-11]. A similar picture is reported from China [12,13]. However, compared to the international literature, few studies with an emphasis on co-infections have been published in China. The majority of the available studies focused on co-infections with *Mycobacterium tuberculosis *and viral hepatitis.

The objectives of the present study were i) to systematically review the peer-reviewed scientific literature on HIV/AIDS, parasites and co-infections published in China between 1986 and 2006; ii) to analyze publication dynamics and classify the identified communications according to research topics; and iii) to highlight neglected research areas.

## Materials and methods

### Literature databases

More than 10 online scientific literature databases are currently available in China, covering the entire spectrum of the Chinese biomedical literature. The three most comprehensive and widely used databases for searching and retrieval of Chinese scientific publications are the China National Knowledge Infrastructure (CNKI), VIP Information (VIP) and Wanfang Database [14,15]. Among them, CNKI is generally acknowledged to cover infectious diseases including HIV/AIDS and parasitic infections most comprehensively [16]. Therefore, the current review focused on CNKI but other databases were also considered. The analysis focused on domestic Chinese literature databases and no international literature repositories or publications by Chinese research groups in international journals were considered. The reason for these restrictions is two-fold: First, the international literature, most notably publications in English, is widely available through international databases such as PubMed, ScienceDirect and ISI Web of Knowledge whereas access to Chinese publications is very limited for non-Chinese speakers. Thus, in our opinion, an analysis of the Chinese-language literature is of special interest to the international scientific community. Second, categorization of publications in international journals often poses problems since funds might originate from outside China and the influence of non-Chinese members of the research group on the choice of research questions is difficult to ascertain.

### Search strategy

References in CNKI, VIP and Wanfang were identified using the following approach. First, scientific publications dealing with HIV/AIDS were identified using the search term  (aizibing/HIV = AIDS/HIV). The search was limited to the period 1986 – 2006, and covered all available journals and publication types. Second, a similar search was performed using the term  (jishengchong = parasite). Third, sub-searches were performed on all publications identified in the search on HIV/AIDS using the Chinese equivalents of 'parasite', '*Pneumocystis*', 'cryptozoite', '*Toxoplasma*', '*Cryptosporidium*', 'amoeba', 'malaria' and '*Leishmania*'.

The retrieved citations were imported into the literature management software EndNote version 9.0 (Thomson Reuters, New York, USA). After manually removing duplicates, comments on previous publications, solicited articles, conference abstracts and notifications, temporal publication patterns were established by stratifying the identified articles by publication year, and detailed analyses of the focus and content of the identified publications were performed. The categories 'case report', 'diagnosis', 'epidemiology', 'health education', 'immunology & molecular research', 'intervention & control', 'reviews', 'clinical therapy & drug resistance' and 'vaccine development' were used to characterize the research field from which individual publications hailed.

## Results

### Publication numbers and temporal patterns

Using identical search terms and limitations for querying the three largest Chinese databases for scientific literature, the highest number of hits resulted from querying CNKI. In this database, a total of 24,511 references dealing with HIV/AIDS and 15,398 publications about parasites were identified in the specified time period. In Wanfang database, 15,925 (HIV/AIDS) and 12,043 (parasites) entries were highlighted, respectively. The least number of hits resulted from querying the VIP database where 13,873 HIV/AIDS-related and 7,068 parasite-specific citations could be identified.

**Figure 1:**
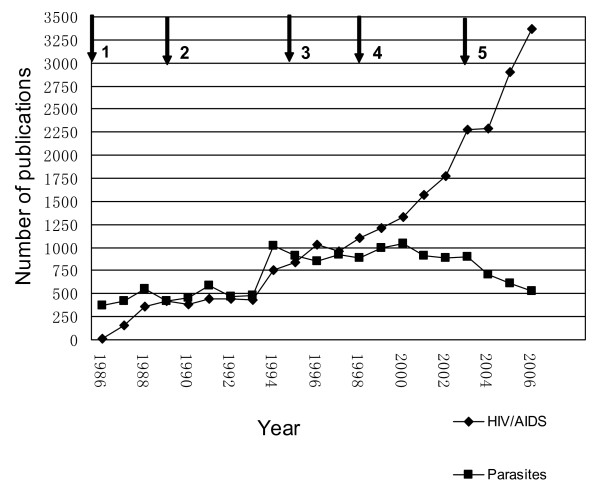
**Annual number of publications focusing on HIV/AIDS and parasites published in Chinese biomedical journals between 1986 and 2006, and major steps in HIV/AIDS policy formulation by the Chinese government**. 1. 1985: First case of HIV/AIDS in China. 2. 1989: 146 HIV-positive cases found among intravenous drug users (IDUs) in Ruili, a border town with Myanmar in Yunnan province, China. 3. 1995: The Ministry of Health publishes the first document urging to step up HIV/AIDS control and prevention. 4. 1998: The State Department publishes the 'Mid- and long term project for HIV/AIDS control and prevention', requiring each level of government to strengthen HIV/AIDS control and prevention activities. 5. 2003: Premier Wen Jia-Bao announces the new national HIV/AIDS control policy "Four Frees and One Care".

Screening the retrieved citations from the CNKI for duplicates, comments etc. resulted in the removal of 486 (HIV/AIDS) and 487 (parasites) references. Hence, a total of 24,025 citations dealing with HIV/AIDS and 14,911 parasite-specific publications were available for further analysis. As shown in Figure 1, the number of publications on HIV/AIDS exponentially increased from 6 publications in 1986 to 3,372 titles in 2006. Meanwhile, the number of parasite-specific publications only gradually and inconsistently increased from 365 titles in 1986 to a maximum of 1,023 publications in 1994. Later, it fluctuated around 900 and started to decline after 2003. In 2006, only 530 publications were recorded in this category. Of note, the publication numbers on HIV/AIDS have remarkable increased after 1995 and 2003, both years when the Chinese government promulgated policies to strengthen HIV/AIDS control and prevention.

### Stratification by research field

The stratification of the identified publications dealing with HIV/AIDS and parasites, respectively, by their research emphasis is displayed in Table 1. According to this classification, the most prominent type of report in the field of HIV/AIDS research were epidemiological investigations: 6,243 (26.0% of the total) fell into this category. The second-largest cluster (2,760 titles or 11.3%) focused on clinical therapy and drug resistance studies. Only 561 or 2.3% of the total number of publications concerned "vaccine development" and 696 titles (2.9%) dealt with "health education". A similar picture emerged from the analysis of the parasite-specific literature where 3,667 titles or 24.6% of the total number of publications reported epidemiological research. Second was "clinical therapy and case reports": 2,201 publications belonged to this class. Literature concerning health education was the least common, representing 0.7% of the total number.

**Table 1:** Stratification of the scientific literature on HIV/AIDS and parasites published in China between 1986 and 2006 by research field.

	**Total**	**Diagnosis**	**Clinical therapy & drug resistance**	**Case reports**	**Vaccine development**	**Epidemiology**	**Health education**	**Interventions and control**	**Others**
HIV/AIDS	24025 (100%)	1229 (5.1%)	2706 (11.3%)	2105 (8.7%)	561 (2.3%)	6243 (26.0%)	696 (2.9%)	1135 (4.7%)	9350 (39.0%)
Parasites	14911 (100%)	1354 (9.1%)	1928 (12.9%)	2201 (14.7%)	132 (0.9%)	3667 (24.6%)	101 (0.7%)	835 (5.6%)	4693 (31.5%)

### HIV/AIDS and parasite co-infections in China

**Figure 2:**
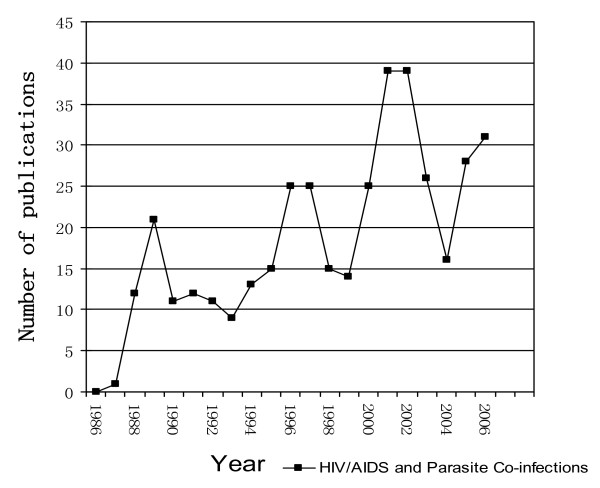
**Annual number of publications on HIV/AIDS and parasite co-infections in Chinese biomedical journals between 1986 and 2006**.

A total of 388 publications dealing with HIV and parasite co-infections were identified among the Chinese scientific literature published between 1986 and 2006, and met the inclusion criteria for further analysis. Figure 2 illustrates the erratic publication patter in this research field. Upon closer examination, a temporal trend towards increased publication activity becomes evident. The number of publications sharply increased from nil in 1986 to 21 in 1989, followed by a certain slack. In both 1996 and 1997, 25 titles were recorded and after another dent, the number of communications rose to the highest number within the study period, i.e. 39 in 2001 and 2002, again followed by a sharp decline. In 2006, the numbers increased again and 31 relevant publications could be identified.

A stratification of these 388 publications on HIV/AIDS and parasite co-infections is displayed in Table 2. HIV/AIDS and *Pneumocystis carinii *co-infection was the single-most researched system, accounting for 30.9% of all publications. The second-most frequently studied associations were those involving *Cryptosporidium *and *Toxoplasma*, accounting for 12.6% and 11.8% of the total number of publications, respectively. To put the number of communications reporting novel research findings into perspective with other types of information generation, it must be noted that almost half of the total number of publications were reviews (48.2%). The most commonly reported original data were case reports which represented 14.4% of all publications, followed by issues related to diagnosis (12.9%) and therapy (10.6%). The smallest category was epidemiological investigations, representing only 6.4% of the identified titles.

**Table 2:** Stratification of publications dealing with HIV/AIDS and parasitic co-infection between 1986 and 2006 by research field.

	Case reports	Diagnosis	Clinical therapy & drug resistance	Reviews	Epidemiology	Immunology &molecular research and others	Total
*P. carinii*	26	31	19	35	4	5	120 (30.9%)
*Cryptosporidium*	2	9	4	25	4	5	49 (12.6%)
*Toxoplasma*	8	2	3	18	6	9	46 (11.8%)
*Plasmodium*	1	1	6	9	2	4	23 (5.9%)
*Leishmania*	3	3	2	8	2	2	20 (5.2%)
*Amoeba*	2	0	0	2	0	1	5 (1.3%)
Other	14	4	7	90	7	3	125 (32.3%)

Total	56 (14.4%)	50 (12.9%)	41 (10.6%)	187 (48.2%)	25 (6.4%)	29 (7.5%)	388 (100%)

Chinese communications on HIV and parasite co-infections focusing on *P. carinii*, *Cryptosporidium *and *Toxoplasma *were analyzed in further detail. The prominence of different research fields varied between the three systems. Among 120 Chinese titles reporting HIV and *P. carinii *co-infections between 1986 and 2006, reviews, diagnosis, case reports, therapy and epidemiological investigations represented 29.2%, 25.8%, 21.7%, 15.8% and 3.3% of the total, respectively. The 49 publications dealing with the HIV-*Cryptosporidium *system fell into the categories reviews, diagnosis, therapy, epidemiological surveys and case reports in decreasing order of prominence. Similarly, the 46 titles on HIV and *Toxoplasma *co-infections comprised reviews as the most prominent category, followed by case reports, epidemiological surveys, therapy and diagnosis.

Overall, the diagnosis of HIV and parasite co-infections represented the largest category of original data and accounted for 12.9% of all publications on HIV-parasite co-infections between 1986 and 2006. Publications dealing with the diagnosis of *P. carinii *among HIV positives were most prominent, accounting for 62% of all titles in this category. The diagnosis of *Cryptosporidium *and *Toxoplasma *co-infections represented 18% and 4%, respectively.

## Discussion

Following the official acknowledgement of local HIV transmission in China by relevant authorities, it soon became evident that the disease was of potentially huge public health significance for the world's most populous country. The Chinese research community started to pay attention to the emerging threat even when the initial neglect and downplay from public institutions still continued, and little means for research were available. Soon after the acknowledgement of the looming crisis by high-ranking officials, resources for research became more readily available, and the topic also caught the attention of the general public. This change in attitudes is well reflected in the evolution of publication numbers. The absolute numbers of publications on HIV/AIDS in China as well as the steep increase over time provide firm evidence that this research field became one of the focus topics of Chinese public health and infectious disease research. It is also likely that the observed trend will continue in the foreseeable future since the challenges posed by HIV/AIDS to public health in China are unlikely to wane any time soon. Thus, it appears highly significant that the government promoted this research field, only after which it started to flourish. China has a tradition of tightly overseeing and steering research and promoting research fields which have been officially identified as top priorities. Thus, the government should continue to foster multi-sectoral and international collaboration and encourage innovation in research [17], without which qualitative improvements in its domestic research capacity will be much slower.

Over the 20-year's history of HIV/AIDS research in China, a diverse approach covering a wide range of basic and applied research questions, from vaccine development through clinical medicine to health education, has developed. The unfolding of the domestic research field partially followed international trends, but there are also differences. In the domestic Chinese literature, the relative contributions of the different research avenues to the overall corpus of HIV/AIDS-specific literature are generally comparable to that of the dedicated international literature [18]. The only major exception is publications dealing with epidemiology which were the most common class in our sample but according to other reviews, clinical therapy is the most common topic in the international literature.

Compared to the dynamic development of HIV/AIDS research, the publication activity on parasitologic topics in China appears more restrained. Over the 20-year period investigated here, a modest increase followed by a stable period and, later, a decreasing trend was found. One explanation might be the ongoing epidemiological transition, i.e. a broader shift from infectious, including parasitic, diseases to chronic non-communicable diseases as the main public health problem in China. This phenomenon can be observed globally in the wake of broad socio-economic development and is arguably more pronounced in China with its rapid development and lifestyle changes over the last 30 years. Viewed from this perspective, it is reasonable that parasitology and parasitic diseases received less attention in relative and even absolute terms after having been controlled in many areas across China. However, parasitic diseases are far from controlled across much of China [6]. Even common parasites like soil-transmitted helminths have not been controlled nationwide. Especially among non-Han ethnic minorities, parasitic diseases still pose a significant public health problem [19,20]. It has also repeatedly been pointed out and recently also been demonstrated that the economic advancement triggered lifestyle and trade pattern changes across the Chinese society and territory which result in the spread, re-emergence or novel appearance of certain previously rare or geographically confined parasitic diseases. This development was highlighted in a report on major human parasitic diseases in China which compared the prevalence of *Clonorchis sinensis *infection in 1990 with that at the beginning of the 21^st ^century. It found that the prevalence had significantly increased in Guangdong and Jilin provinces as well as Guangxi Zhuang Autonomous Region, with reported increases of 182%, 630% and 164%, respectively [21]. Recently, the emergence of yet another parasite in China has been reported, i.e. *Angiostrongylus cantonensis *[22]. Thus, continued attention must be paid to parasitic diseases across China, especially in the fields of health education, control and surveillance [23]. This need for continuing research contrasts with the apparent decline in publication activities on parasitological topics, and indicates that efforts might be required to strengthen domestic parasitological research. However, it can not be ruled out that the observed decline in publication activity in China in the field of parasitology is only one side of a fundamental shift where high-quality studies are increasingly being published in English in international journals while less sophisticated research is being confined to local outlets. This would indicate not a decline in parasitological studies but rather improved quality and awareness for the global relevance of research findings generated in China by Chinese researchers. To fully understand this aspect of the evolution of scientific research, the question should be addressed through a thorough analysis of not only the Chinese literature published in China but also communications by China-based research groups in international journals. In a next step, the authors plan to expand the present study to also include such publications, complemented by a comparison between publication patterns and the perceived quality of communications in national Chinese journals and in international English-language outlets.

In HIV/AIDS research, high numbers of studies were and continue to be carried out in China by local researchers and research consortia, and many interesting results were obtained. However, most research findings were published in domestic journals only available in Chinese, thus greatly hampering the exchange with the international scientific community. For most non-Chinese researchers the Chinese literature is not accessible due to language and technology barriers (and vice-versa, a large part of the Chinese research community operates in ignorance of scientific activities beyond China), incurring scientific and economic costs which await quantification but might be substantial. Some issues of concern are study duplications and hence uneconomic resource allocation, and missed potential for significant insights which can result from integrating findings originating from different studies conducted at various locations and under different conditions. Especially in rural areas, where local healthcare professionals generate a huge amount of relevant data, a notable shortage of funds, infrastructure and training as well as communication skills hamper improvements in the quality of scientific research. The most that can usually be generated under such circumstances are studies on clinical diagnosis, treatment and individual case reports. It has previously been concluded that the domestic Chinese literature were a huge treasure trove which should be explored and opened up to the international community [16,24,25]. It remains to be seen if this could also yield significant insights and advances in the field of HIV/AIDS research as it has done in other domains, e.g. schistosomiasis [26].

The number of publications dealing with HIV/AIDS and parasite co-infections published in China is limited. The majority of these articles are reviews (often of studies conducted outside China) and case reports. Even basic research questions still await a definitive answer in China. For example, there are no comprehensive data on the overall prevalence of different parasitic infections among AIDS patient in China. This is especially troubling if seen against the backdrop of the high fraction of HIV infections stemming from poor rural regions of China, and the wide variety of parasites which still thrive in these areas. A distinct subgroup among the global HIV cohort which is prominent in China is commercial blood donors. In China, HIV infections among blood donors occurred at least up to 1994 due to risky procedures, one of the worst public health scandals of modern China [27]. The majority of these cases are concentrated in the remote countryside of Henan and Anhui province, and most patients with AIDS symptoms urgently need antiviral as well as antiparasitic treatment. It has been reported that among them, parasite infections were a major cause of death [28-30]. The fluctuating numbers of publications on HIV/AIDS and parasite co-infections suggested that only few groups focused on this research field. Most groups working in HIV/AIDS research lack parasitological knowledge and neglect the impact of parasites on the progress of AIDS. As studies showed, parasitic infections could impact on the immune system, and timely treatment of infections might influence the progress of AIDS. Parasitologists, on the other hand, are often short of the necessary qualifications and technical as well as monetary means to perform research connected to HIV/AIDS. There also is a general shortage of knowledge regarding the importance and diagnosis of protozoa. The available epidemiological surveys were mainly carried out by clinical doctors and little basic research is being done in this avenue. Thus, more collaboration between HIV/AIDS research and parasitology is urgently needed. Studies focusing on the prevalence and significance of parasite infections among HIV infected individuals in China are scarce but highly relevant. Forthcoming results could contribute to the initiation of timely and appropriate therapies, thus reducing morbidity and mortality, and improving the quality of life of the affected individuals. This could also further contribute to the development of a sound strategy for HIV/AIDS prevention and control in China and beyond.

## Conclusion

HIV/AIDS research in China has ballooned over the last 20 years following focused government attention and steep increases in funding. By contrast, parasitology lost ground in relative but also absolute terms, and publication numbers are declining. The structure of the published research suggests a predominance of descriptive and repetitive studies, with relatively little innovation. The large number of publications only available in Chinese suggests missed chances for scientific advancement due to ignorance of research results and duplication of studies, indicating an uneconomic use of overall research funding. Research on parasitology in general and on HIV/AIDS and parasite co-infections in particular should be strengthened in order to better reflect the public health relevance of the respective infections. The exchange between the Chinese and the international research community should be promoted by encouraging the publication of significant findings by Chinese scientists in international journals, the publication of major review articles in the respective other language, and by the promotion of English abstracts and articles in Chinese scientific journals and their improved accessibility for foreign researchers.

## List of abbreviations

AIDS: Acquired Immunodeficiency Syndrome; CNKI: China National Knowledge Infrastructure (a literature database); HIV: Human Immunodeficiency Virus; VCT: Voluntary Counseling and Testing; VIP: VIP Information (a literature database).

## Competing interests

The authors declare that they have no competing interests.

## Authors' contributions

LGT conceived the study, carried out data collection and analysis and drafted the manuscript. PS conceived the project, assisted in the interpretation of the results and revised the manuscript. JXC and SHC conceived the project and provided technical support for data collection and analysis. XNZ conceived the study and revised the manuscript. All authors read and approved the final manuscript.
